# Tau protein mediates the association between frailty and postoperative delirium: a machine learning model incorporating cerebrospinal fluid biomarkers

**DOI:** 10.3389/fneur.2025.1608264

**Published:** 2025-09-17

**Authors:** Yizhi Liang, Chuanlin Mu, Wenjie Kong, Kun Wang, Shuhui Hua, Yuanlong Wang, Xia Liu, Hongyan Gong, Yanan Lin, Chuan Li, Xu Lin, Yanlin Bi, Bin Wang

**Affiliations:** ^1^Department of Anesthesiology, Qingdao Municipal Hospital, Qingdao, China; ^2^The Second School of Clinical Medicine, Binzhou Medical University, Yantai, China; ^3^Department of Anesthesiology, Shandong Second Medical University, Weifang, China

**Keywords:** machine learning, postoperative delirium, cerebrospinal fluid, surgery, confusion matrix

## Abstract

**Objective:**

Postoperative delirium (POD) is a prevalent neurological complication linked to adverse clinical outcomes. The underlying mechanisms of POD remain unclear. This study aimed to investigate the association between POD and frailty and determine whether frailty influences POD incidence. Furthermore, machine learning algorithms were utilized to identify key predictors of POD in patients undergoing hip or knee replacement.

**Methods:**

A total of 625 Han Chinese patients were recruited between September 2021 and May 2023. Preoperative frailty was assessed using the Frailty Scale and Frailty Phenotype criteria. The Mini-Mental State Examination (MMSE) evaluated preoperative cognitive function, while the Confusion Assessment Method (CAM) diagnosed POD. The severity of POD was additionally quantified using the Memorial Delirium Assessment Scale (MDAS). Receiver Operating Characteristic (ROC) curve analysis explored the association between preoperative frailty and POD, and the mediating effect of cerebrospinal fluid (CSF) biomarkers was analyzed. Ten machine learning algorithms—including Logistic Regression (LR), Support Vector Machine (SVM), Gradient Boosting Machine (GBM), Artificial Neural Network (ANN), Random Forest (RF), XGBoost, K-Nearest Neighbors (KNN), AdaBoost, LightGBM, and CatBoost—were implemented to develop predictive models. The dataset was randomly split into training (70%) and testing (30%) subsets. Ten-fold cross-validation was incorporated during model training and validation to mitigate overfitting and enhance generalizability. Model performance was evaluated using multiple metrics, such as accuracy, sensitivity, specificity, precision, Brier score, area under the ROC curve (AUC), and F1 score. Furthermore, graphical analyses—including calibration curves, decision diagrams, clinical impact curves, and confusion matrices—were applied to assess model robustness and clinical utility. Finally, SHAP (Shapley Additive Explanations) analysis elucidated the model’s decision-making process, emphasizing the pivotal role of preoperative frailty in POD prediction.

**Results:**

The incidence of POD was 14.7%. The study identified frailty, Tau, and P-tau as significant risk factors for POD (OR = 67.229, 95% CI: 34.649–130.444, *p* < 0.001; OR = 1.020, 95% CI: 1.016–1.024, *p* < 0.001; OR = 1.018, 95% CI: 1.010–1.027, *p* < 0.001). ROC curve analysis (AUC = 0.983) demonstrated that combining frailty with CSF biomarkers had strong predictive power for distinguishing POD. The direct effect of frailty on POD was 0.504878, the total effect was 0.6547619, and the mediating effect of Tau accounted for 22.89%. Using Lasso regression for variable selection, we subsequently identified eight predictors—frailty, Tau, Aβ42/Tau, Aβ40, age, Aβ42, P-tau, and drinking history—from the training set via logistic regression. Based on these factors, we constructed 10 machine learning models. Among all machine learning algorithms, GBM performed the best, achieving an AUC of 0.973 (95% CI, 0.973–1.000) in the test set. Furthermore, SHAP analysis confirmed that frailty and Tau were the key determinants influencing the machine learning model’s predictions.

**Conclusion:**

Preoperative frailty is an independent risk factor for POD. A machine learning model for predicting POD in patients undergoing hip or knee replacement was developed, with GBM demonstrating superior performance among all models. The GBM-based model enabled early identification of patients at high risk of delirium.

## Introduction

POD is an acute disturbance in attention and cognition, primarily observed in older adults. It is associated with severe outcomes, high healthcare costs, underdiagnosis, and increased mortality risk (1). As life expectancy increases, the number of older surgical patients has risen correspondingly. POD is the most common postoperative complication in this population, with an incidence ranging from 15 to 25% (2). Currently, the pathophysiological mechanisms of delirium remain unclear and may involve multiple distinct pathways (3).

While the precise origins of POD remain unclear, emerging evidence suggests that blood–brain barrier dysfunction, CSF biomarker alterations, and neuroinflammatory cascades are critical contributors to POD pathogenesis (4). In a healthy brain, tau protein performs essential cellular functions, including binding to tubulin to promote microtubule polymerization, stabilizing microtubule architecture, preventing tubulin dissociation, and facilitating microtubule bundle formation (5). Under pathological conditions, abnormal phosphorylation of tau disrupts its ability to assemble microtubules and maintain structural integrity, severely compromising its interaction with tubulin. Hyperphosphorylated tau not only competes with normal tau for tubulin binding but also adheres to other microtubule-associated proteins, displacing them from microtubules. This leads to microtubule depolymerization and network destabilization, ultimately causing microtubule breakdown and structural collapse. Concurrently, P-tau aggregates into paired helical filaments (PHFs) and neurofibrillary tangles (NFTs) (6). Research suggests that tau protein strongly predicts the occurrence of POD in older adults with undiagnosed dementia, and it likely plays a significant role in the development of POD (7). Beyond tau protein, the amyloid-beta (Aβ) pathway is another central component of Alzheimer’s disease pathophysiology (8). Aβ peptides, particularly Aβ42 and Aβ40, are derived from the sequential proteolytic cleavage of the amyloid precursor protein (APP). Aβ42 is highly aggregation-prone and is the primary component of amyloid plaques, a hallmark of AD neuropathology. The ratio of Aβ42 to Aβ40 (Aβ42/Aβ40) in cerebrospinal fluid (CSF) is a sensitive indicator of brain amyloid deposition, typically showing a decrease due to the sequestration of Aβ42 into plaques (9).

Frailty is defined as a reduced ability to restore homeostasis after a stressor, significantly elevating the risk of adverse outcomes such as falls, delirium, cognitive impairment, and disability (10). Existing evidence suggests that the prevalence of frailty escalates with advancing age in older adults (11). Frailty is associated with both the neuropathological hallmarks of Alzheimer’s disease (AD) and clinical manifestations such as cognitive decline and dementia. Importantly, frailty and Alzheimer’s disease dementia share overlapping risk factors and clinical features, including age, chronic inflammation, functional impairment, and atypical disease presentations (12–14). Given the analogous neuropathological processes between POD and AD (15), this study examines the relationship between frailty and POD. Although extensive literature has documented this association, the underlying mechanism remains elusive.

In recent years, machine learning (ML) has emerged as a promising method for building predictive models, with significant potential and prospects for its application in healthcare (16). Machine learning algorithms have shown remarkable accuracy in predictions (17). While a number of delirium risk factors have been leveraged to build predictive models for diverse patient groups, we are still lacking a tailored model designed specifically to forecast delirium in frail patients (18).

Therefore, our objectives are two folds: (1) exploring the association between frailty and postoperative delirium, and (2) developing a machine learning-based predictive model for postoperative delirium that uses frailty along with other clinical features to predict the occurrence of delirium in patients. The model has been visualized and further promoted for clinical application, aiming to assist healthcare providers in the early identification of high-risk patients, minimize postoperative delirium among older patients and enhance overall patient outcomes.

## Materials and methods

### PNDABLE database

Our study drew participants from the PNDABLE database, a long-term cohort study that’s currently investigating the risk factors and biomarkers associated with perioperative neurocognitive disorders (PND). This study focuses on a Han Chinese population, aged 40 to 90, residing in northern China. The Ethics Committee at Qingdao Municipal Hospital gave the thumbs up to our study protocol. What’s more, we are officially registered with the Chinese Clinical Trial Registry under the registration number ChiCTR2000033439.

Participants were fully informed about the research goals and methodology, and their written consent was obtained. They were free to withdraw from the study at any time without facing any negative consequences. The CSF biomarkers and blood samples collected will be stored for future scientific investigation.

### Participants

This study was carried out at Qingdao Municipal Hospital, drawing upon information from the PNDABLE database, which was collected between September 2021 and May 2023. Participants were chosen according to predefined eligibility standards. The criteria for inclusion required: (1) individuals between 40 and 90 years old; (2) those scheduled for hip or knee replacement surgery; (3) patients undergoing combined spinal and epidural anesthesia; (4) classification within ASA grades I–III based on the guidelines of the American Society of Anesthesiologists (ASA); (5) individuals who willingly agreed to join the study and signed a consent form; and (6) those exhibiting normal cognitive function before the operation without any communication difficulties. The exclusion conditions comprised: (1) serious neurological conditions, including epilepsy, multiple sclerosis, or infections of the central nervous system; (2) severe psychiatric disorders such as major depression or delirium; (3) significant impairments in vision or hearing; (4) abnormal blood coagulation detected before surgery; and (5) a MMSE score lower than 24 in the preoperative evaluation.

### Neuropsychological testing

The Confusion Assessment Method (CAM) was used in these evaluations to identify the presence of POD. Individuals who had positive delirium tests were placed in the POD group, while those with negative results were classified as the non-POD (NPOD) group (19). Furthermore, The MDAS was used to evaluate the severity of POD (20). For all participants, this assessment was performed 1 to 7 days postoperatively by trained medical professionals who were unaware of the patient’s perioperative condition. When the assessor was unavailable, delirium was determined based on electronic medical records and nursing documentation.

### Anesthesia and surgery

Each participant underwent a scheduled surgical procedure under combined spinal-epidural anesthesia. Prior to surgery, no preoperative medication was administered, and patients adhered to fasting requirements, abstaining from food for 8 hours and liquids for 4 hours. Upon arrival in the operating theater, standard physiological monitoring was implemented, including electrocardiography, arterial blood pressure tracking, oxygen saturation measurement, and intravenous line placement. An experienced anesthesiologist carried out the lumbar puncture at the L3-L4 intervertebral space. After the needle was properly positioned, 2 to 2.5 mL of 0.66% ropivacaine was put in over the course of 30 s after 2 mL of CSF fluid was taken for biomarker examination. The level of anesthesia was carefully regulated, ensuring it did not exceed T8. Throughout the operation, vital signs such as heart rate, blood pressure, and oxygen levels were recorded at three-minute intervals, with supplemental oxygen provided at 5 L/min via a facemask. After surgery, patients were observed in the post-anesthesia care unit (PACU) for 30 min before being returned to their respective wards once their vital signs stabilized.

### CSF core biomarkers measurements and collection

A 2 mL CSF specimen was obtained in a polypropylene centrifuge tube, then centrifuged at ambient temperature for 10 min (2000 × g). The supernatant obtained was gently removed and stored in an enzyme-free Eppendorf (EP) tube at −80 °C for future experimental use. Biomarkers such as Aβ42, Aβ40, P-tau, and total tau were analyzed using enzyme-linked immunosorbent assay (ELISA) kits (Thermo Scientific, Multiskan MK3) following the guidelines provided by the manufacturer. To eliminate any risk of bias, laboratory personnel conducting the assays were blinded to all associated clinical information.

### Sample size estimation

Based on preliminary analysis, five covariates were identified for potential inclusion in the logistic regression model. To guarantee adequate statistical power to meet the study’s objectives, a sample size of 625 participants was calculated, assuming a 20% discontinuation rate and a 15% occurrence of POD.

### Frailty

Regarding the assessment of frailty, we use internationally recognized methods for the rapid identification of frailty, namely the Frailty Phenotype and the Frailty Scale. The Frailty Phenotype assesses five manifestations of frailty: (1) Weight loss: Unintentional weight loss ≥10 lbs. in the previous year. (2) Weakness: Determined by grip strength measurement, with gender- and body mass index (BMI)-adjusted cutoffs. (3) Exhaustion: Assessed by two items from the modified 10-item Center for Epidemiologic Studies Depression (CES-D) scale. (4) Low physical activity: Evaluated using the modified Minnesota Leisure Time Activity Questionnaire. (5) Slowed walking speed: >7 s to complete a 4-meter walk test. The Frailty Scale evaluates frailty based on five criteria: (1) Fatigue: Subjective feeling of being exhausted “most or all of the time” during the past 4 weeks. (2) Reduced endurance: Inability to climb one flight of stairs without assistive devices or resting. (3) Mobility limitation: Difficulty walking two city blocks (approximately 200–300 meters) independently. (4) Multimorbidity: Presence of five or more comorbidities from the following list: hypertension, diabetes mellitus, cancer, asthma, chronic pulmonary disease, coronary artery disease, congestive heart failure, angina, arthritis, stroke, and chronic kidney disease. (5) Weight loss: Unintentional weight loss exceeding 5% of body weight during the past year (21). The frailty assessment criteria are defined as follows (1 point for each item met, total score interpretation, 0 = robust, 1–2 = pre-frail, ≥3 = frail). If any one of these methods meets three or more of the symptoms, frailty is considered present.

### Statistical analysis

The data’s normality was evaluated using the Kolmogorov–Smirnov test. The mean ± standard deviation (SD) was used to represent data that had a normal distribution. Continuous variables exhibiting non-normal distributions (assessed via Kolmogorov–Smirnov test), such as age, duration of anesthesia, and surgery duration, were summarized as median and interquartile range (IQR). Categorical variables, including sex and frailty status, were summarized as frequency counts and percentages. Frequency counts and percentages were used to report categorical information. Statistical significance between groups was determined via Wilcoxon tests (continuous data) and Chi-square analyses (categorical data).

Firstly, Feature selection for postoperative delirium (POD)-related factors was performed using LASSO regression. To control for potential confounders, we conducted multiple sensitivity analyses, creating three distinct correction models: (1) age (50–90 years), sex, years of education, and MMSE scores; (2) age (50–90 years), sex, years of education, MMSE scores, smoking and drinking history, and diabetes; and (3) age ≥65, sex, years of education, MMSE scores, smoking and drinking history, and diabetes.

Secondly, the predictive capabilities of frailty and the combination of frailty with CSF biomarkers for POD were assessed; the ROC curve was employed for this purpose.

Thirdly, the aim was to explore the potential mediating role of CSF biomarkers (Aβ42, Aβ40, Tau, P-tau, Aβ42/Tau, Aβ42/P-tau) in the observed association between frailty and POD. To this end, 10,000 iterative analyses were performed using Stata MP17.0.

Finally, In order to identify which variables were part of the delirium prediction model, this study used univariate logistic regressionin in the test set. To achieve optimal prediction, 10 models were constructed for the experiment, including LR, SVM, GBM, NN, RF, XGBoost, KNN, AdaBoost, LightGBM, and CatBoost. Each algorithm has its unique strengths, making it suitable for different data structures and task requirements. Ten-fold cross-validation on the training dataset was used to choose these models’ hyperparameters. Cross-validation evaluates model performance more effectively by averaging results across multiple trials. The performance of the models was assessed using metrics such as AUC, Brier score, accuracy, sensitivity, specificity, precision, and F1 score. To improve the clarity and understanding of the models, we employed SHAP analysis. This method clarifies predictive results, details individual feature importance, and provides practical guidance.

All computations were performed using IBM SPSS software version 27, visualizations were created utilizing R software version 4.3.3, and data analysis done utilizing using Stata MP17.0. *p* < 0.05 indicated the level of significance.

## Results

### Participant characteristics

**Figure 1:**
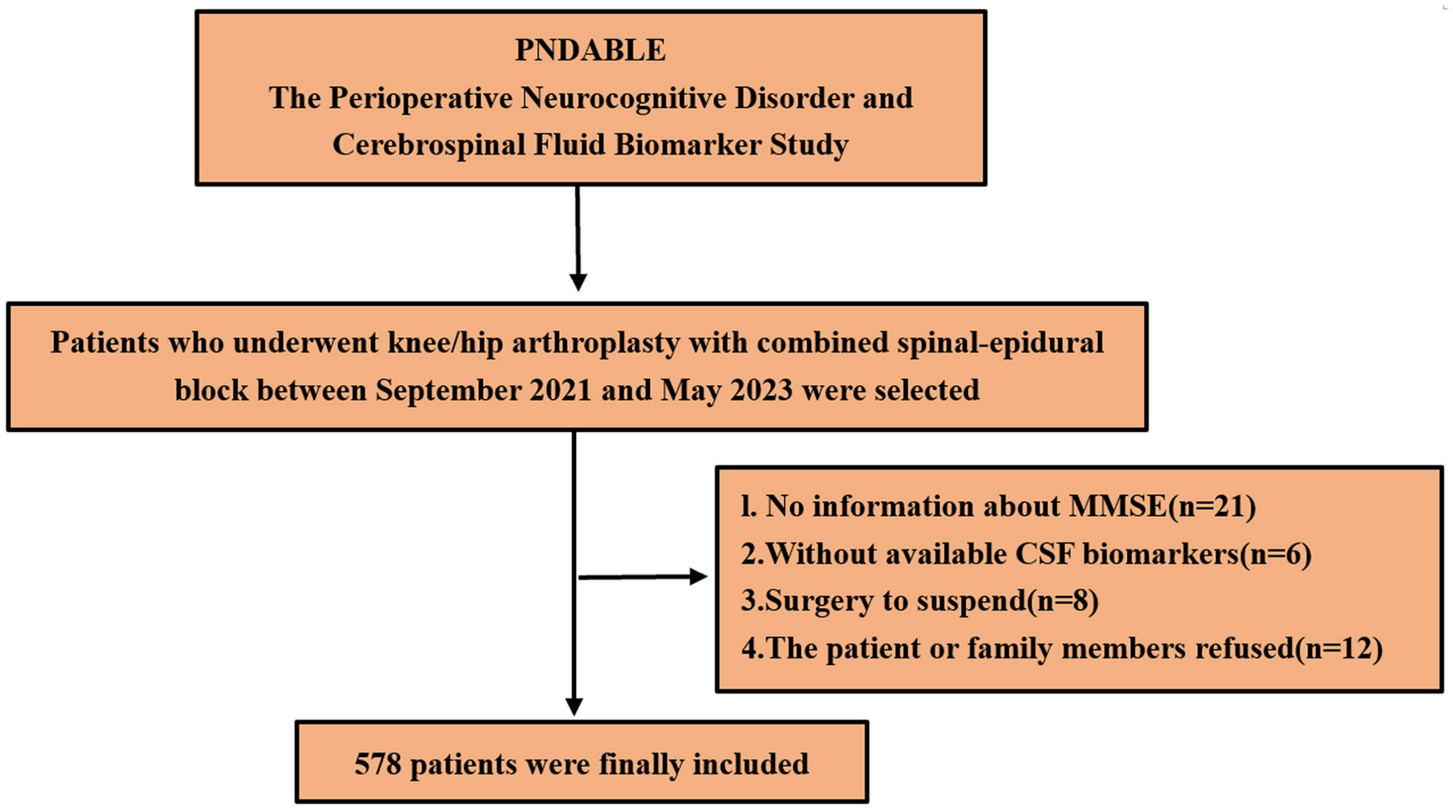
Flowchart of study cohort recruitment.

We eventually included 578 patients for statistical analysis (Figure 1). POD incidence of 14.7% (n = 85 of the 578 patients) and 70 patients with frailty in the POD group was observed. Compared to the NPOD group, the POD group showed significant differences in CSF biomarkers (including Aβ42, Aβ40, P-tau, Tau, Aβ42/Tau, and Aβ42/P-tau) (*p* < 0.05). There were statistically significant differences in age, frailty, BMI, ASA, and MDAS score, (*p* < 0.05). No significant differences were found in VAS scores, Education, Sex, Somking history, Drinking history, Coronary heart disease, Diabetes, MMSE scores, Duration of anesthesia, Duration of surgery, Intraoperative volume of infusion and Intraoperative blood loss between the two groups (*p* > 0.05) (Table 1).

**Table 1:** Patient characteristics and baseline variables.

Characteristic	NPOD (*n* = 493)	POD (*n* = 85)	*p*
Age [year, M(Q)]	63.23 (13)	72.69 (17.5)	<0.001*
Sex [*n* (%)] Man	248 (50.3)	44 (51.80)	0.804
Women	245 (49.7)	41 (48.20)	
BMI [M(Q)]	25.77 (4.51)	24.49 (5.26)	0.014*
ASA [*n* (%)] I	45 (9.1)	5 (5.9)	<0.001*
II	411 (83.4)	60 (70.6)	
III	37 (7.5)	20 (23.5)	
Education [year, M(Q)]	8.88 (6)	8.41 (7.00)	0.275
Smoking history [*n* (%)]	18 (3.7)	3 (3.5)	0.914
Drinking history [*n* (%)]	4 (0.8)	3 (3.5)	0.083
Coronary heart disease [*n* (%)]	45 (9.1)	8 (9.4)	0.933
Diabetes [*n* (%)]	81 (16.4)	16 (18.80)	0.586
VAS score[M(Q)]	0.35 (1.0)	0.31 (0)	0.313
MDAS [scores, M(Q)]	3.39 (6)	14.58 (5)	<0.001*
MMSE [scores, M(Q)]	27.24 (4)	26.93 (3.00)	0.136
Frailty [*n* (%)]	32 (6.5)	70 (82.4)	<0.001*
Aβ40 [pg/ml, M(Q)]	5633.20 (2445)	6742.84 (2695.5)	<0.001*
Aβ42 [pg/ml, M(Q)]	618.62 (269.45)	402.19 (351.25)	<0.001*
P-tau [pg/ml, M(Q)]	46.95 (18.37)	60.60 (34.64)	<0.001*
Tau [pg/ml, M(Q)]	191.15 (99.7)	367.92 (82.58)	<0.001*
Aβ42/P-tau [M(Q)]	14.67 (7.20)	7.99 (8.18)	<0.001*
Aβ42/T-tau [M(Q)]	3.71 (2.09)	1.24 (1.17)	<0.001*
Duration of anesthesia (min)	145.40 (65.00)	142.18 (72.5)	0.752
Duration of surgery (min)	96.79 (55.00)	91.47 (55.00)	0.444
Intraoperative volume of infusion (ml)	1167.91 (600.00)	1184.94 (500)	0.426
Intraoperative blood loss (ml)	112.58 (190.00)	112.36 (180.00)	0.464

POD, postoperative delirium; NPOD, non-postoperative delirium; MDAS, Memory Delirium Assessment Scale; ASA, American Society of Anesthesiologists Physical Status Classification. Aβ42, amyloid beta42; P-tau, phosphorylated Tau.

Continuous variable use Student’s *t* test or Mann–Whitney U, Categorical variable use Chi-square test.

Non-normally distributed numerical variables are statistically described by Median (Interquartile spacing) [M(Q)].

Categorical variables are statistically described by Sample size (Percent) [*n* (%)].

*p < 0.05.

### Results of binary logistic regression and sensitivity analysis

Binary logistic regression analysis indicated that frailty (OR = 67.229, 95% CI 34.649–130.444, *p* < 0.001), Tau (OR = 1.020, 95% CI 1.016–1.024, *p* < 0.001), and P-tau (OR = 1.018, 95% CI 1.010–1.027, *p* < 0.001) were identified as significant risk factors for POD. Conversely, CSF levels of Aβ42, Aβ42/Tau, and Aβ42/P-tau were protective factors against POD (OR = 0.994, 95% CI 0.993–0.996, *p* < 0.001; OR = 0.140, 95% CI 0.094–0.21, *p* < 0.001; OR = 0.785, 95% CI 0.742–0.831, *p* < 0.001). We conducted a sensitivity analysis to enhance the credibility of our findings by accounting for various confounding factors, including age, sex, years of education, MMSE scores, Drinking history, and Smoking history, as well as the presence of diabetes. It was concluded that this analysis hardly affected the results (Table 2).

**Table 2:** Logistic regression analysis of factors influencing POD.

Characteristic	Model 1^a^		Model 2^b^		Model 3^c^		Model 4^d^	
	OR (95% CI)	*p*	OR (95% CI)	*p*	OR (95% CI)	*p*	OR (95% CI)	*p*
Frailty	67.229 (34.649–130.444)	<0.001*	81.938 (36.643–183.219)	<0.001*	95.278 (40.862–222.164)	<0.001*	50.306 (18.691–135.396)	<0.001*
Aβ40, pg/ml	1.000 (1.000–1.001)	<0.001*	1.000 (1.000–1.001)	<0.001*	1.000 (1.000–1.001)	<0.001*	1.000 (1.000–1.001)	<0.001*
Tau, pg/ml	1.020 (1.016–1.024)	<0.001*	1.019 (1.015–1.024)	<0.001*	1.020 (1.016–1.025)	<0.001*	1.017 (1.012–1.022)	<0.001*
Aβ42, pg/ml	0.994 (0.993–0.996)	<0.001*	0.994 (0.992–0.996)	<0.001*	0.994 (0.992–0.996)	<0.001*	0.995 (0.993–0.997)	<0.001*
P-tau, pg/ml	1.018 (1.010–1.027)	<0.001*	1.017 (1.008–1.026)	<0.001*	1.018 (1.008–1.027)	<0.001*	1.014 (1.003–1.026)	0.016*
Aβ42/P-tau	0.785 (0.742–0.831)	<0.001*	0.785 (0.737–0.836)	<0.001*	0.784 (0.736–0.835)	<0.001*	0.818 (0.759–0.882)	<0.001*
Aβ42/Tau	0.140 (0.094–0.21)	<0.001*	0.142 (0.091–0.221)	<0.001*	0.139 (0.089–0.218)	<0.001*	0.184 (0.111–0.306)	<0.001*

OR, odds ratio; 95% CI, 95% confidence interval; Aβ42, β-amyloid42; P-tau, phosphorylated tau.

a Model 1: Unadjusted.

b Model 2: Adjusted for age (50–90), sex, years of education and MMSE scores.

c Model 3: Adjusted for age (50–90), sex, years of education, MMSE scores, smoking history, drinking history, and diabetes.

d Model 4: Adjusted for age≥65, sex, years of education, MMSE scores, smoking history, drinking history, and diabetes.

*p < 0.05.

### Causal mediation analyses

**Figure 2:**
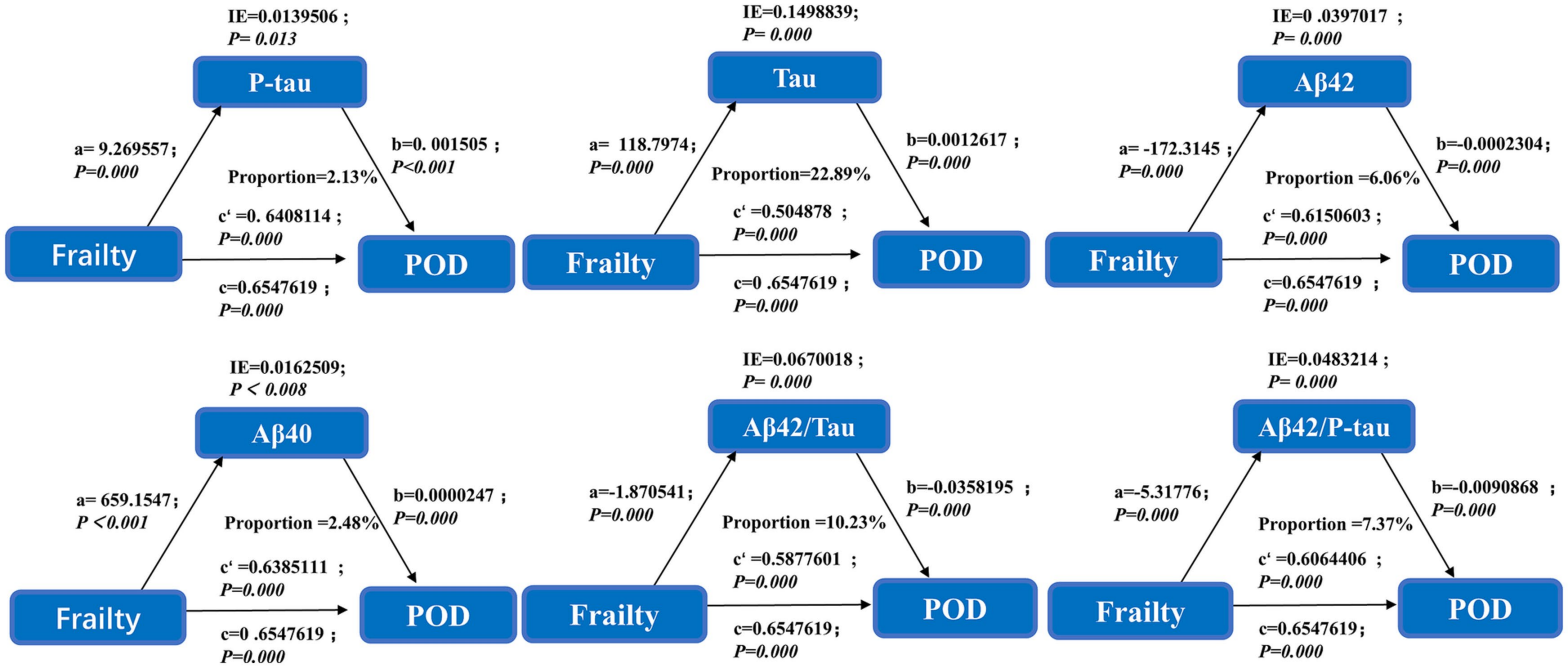
This mediation effect highlights Tau as a potential biomarker bridging frailty and POD pathogenesis.

Mediation analysis suggested that Tau may partially explain the observed association between frailty and POD, accounting for 22.89% of the association (Figure 2).

### Preoperative frailty and postoperative delirium

**Figure 3:**
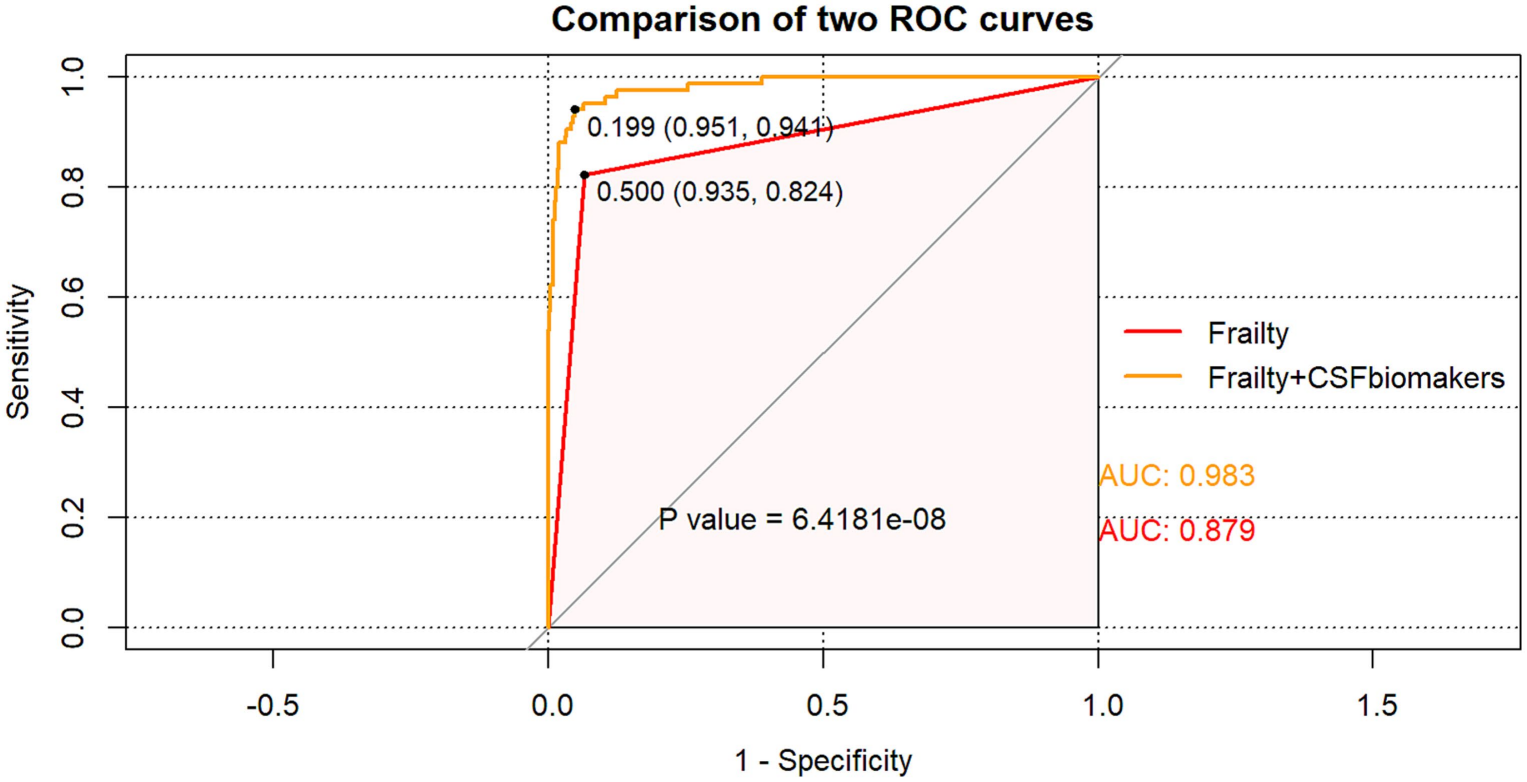
The ROC curve analysis of frailty, and the combination of frailty and CSF biomarkers showed that the combination of frailty and CSF biomarkers had a high diagnostic value for POD.

In order to assess the accuracy of frailty and CSF biomarkers in predicting POD following surgery, we constructed a ROC curve and AUC, shows the prediction of postoperative delirium by different risk indicators. Frailty with CSF biomarkers has the largest AUC area than Frailty (AUC = 0.983: AUC = 0.879), which offers significant insights into the clinical recommendations for preoperative frailty screening and CSF biomarker measurement (Figure 3). The NRI (continue) (1.462, 95% CI 1.299–1.626 *p* < 0.05) also indicates that the combination of frailty and biomarkers performs better.

### Establishment of prediction models

**Figure 4:**
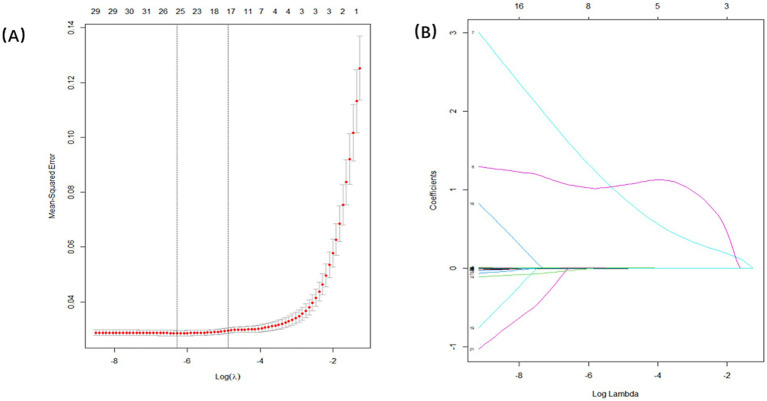
Demographic and clinical feature selection using the LASSO regression. **(A)** Tuning parameter selection in the Lasso model used 100-fold cross-validation. **(B)** Lasso coefficients produced by the regression analysis, as shown in **A**.

**Figure 5:**
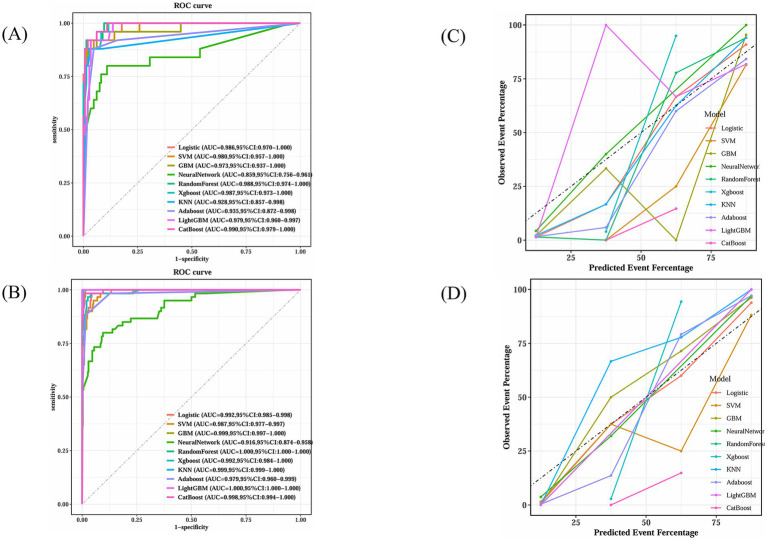
GBM’s superior calibration indicates its reliability in clinical prediction scenarios. **(A)** Receiver operating characteristic curves for ten machine learning models in testing dataset. **(B)** Receiver operating characteristic curves for ten machine learning models in training dataset. **(C)** Calibration plot for ten machine learning models in testing dataset. **(D)** Calibration plot for ten machine learning models in training dataset.

The dataset, comprising 578 entries, was randomly split into a training group and a test group at a 7:3 ratio, with 406 individuals in the training cohort and 172 in the testing cohort. The training group’s data were analyzed to determine essential factors associated with POD and to develop predictive models. The optimal parameter (lambda) in the LASSO model was selected through 100-fold cross-validation. Dotted reference lines were plotted at the optimal values determined by both the minimum criteria and the 1 standard error (1-SE) criteria. A vertical line was drawn at the value selected via 100-fold cross-validation (Figure 4). After LASSO regression variables selected (Table 3), the following predictors were selected through logistic regression analysis including frailty, Tau, Aβ42/Tau, Aβ40, age, Aβ42, P-tau, and drink (Table 4). The test set data were used to validate the predictive models. Using the aforementioned eight variables, 10 predictive models were constructed to predict delirium in frail patients. The ROC curves for the 10 predictive models are shown in Figures 5A,B, with the GBM predictive model achieving the high AUROC. Additionally, 10-fold cross-validation was conducted to assess the reliability of the predictive models, with the GBM model showing the best performance. The calibration curve indicated that the GBM model had good calibration (Figures 5C,D), indicating the model’s strong predictive accuracy.

**Table 3:** LASSO regression of important variables related to POD.

Variables	Coefficient	Lambda.min
Aβ42	−0.000198	0.002470353
Aβ40	0.000011	
Tau	0.000842	
P-tau	0.000252	
Aβ42/ Tau	0.014016	
MDAS	0.033708	
Frailty	0.262324	
Intraoperative blood loss	−0.000072	
VAS score	0.004584	
Age	0.001461	
Education	−0.002948	
SEX	−0.000876	
Diabetes	−0.010697	
Coronary heart disease	−0.000955	
Drinking history	0.115089	
Smoking history	−0.006768	
BMI	−0.001030	

**Table 4:** Logistic univariate regression of training set.

Variables	Univariate analysis OR (95% CI)	*p*
Age	1.10 (1.06–1.14)	<0.001
Sex man	1.23 (0.71–2.15)	0.460
Women		
BMI [M(Q)]	0.95 (0.88–1.02)	0.172
Education [year, M(Q)]	1.00 (0.93–1.07)	0.938
Smoking history [*n* (%)]	1.25 (0.35–4.48)	0.734
Drinking history [*n* (%)]	9.05 (1.48–55.37)	0.017
Coronary heart disease [*n* (%)]	0.92 (0.34–2.48)	0.875
Diabetes [*n* (%)]	0.88 (0.41–1.88)	0.734
VAS score[M(Q)]	1.06 (0.67–1.66)	0.805
MDAS [scores, M(Q)]	837336743.23 (0.00-Inf)	0.966
MMSE [scores, M(Q)]	0.95 (0.83–1.09)	0.457
Frailty [*n* (%)]	86.25 (37.84–195.68)	<0.001
Aβ40 [pg/ml, M(Q)]	1.00 (1.00–1.00)	<0.001
Aβ42 [pg/ml, M(Q)]	0.99 (0.99–1.00)	<0.001
P-tau [pg/ml, M(Q)]	1.02 (1.01–1.03)	<0.001
Tau [pg/ml, M(Q)]	1.02 (1.02–1.03)	<0.001
Aβ42/T-tau [M(Q)]	0.14 (0.08–0.22)	<0.001
Intraoperative blood loss (ml)	1.00 (1.00–1.00)	0.720

**Figure 6:**
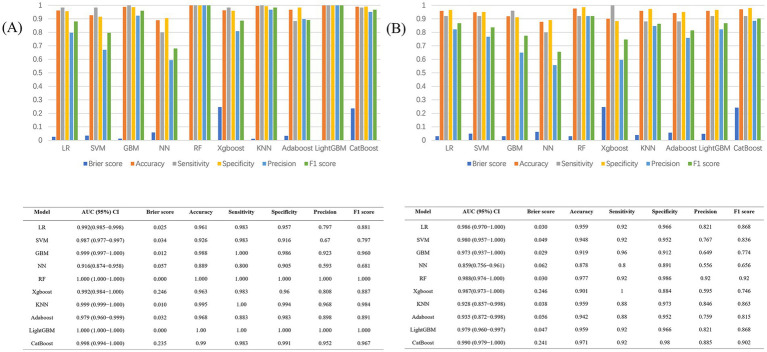
**(A)** Performance metrics of the 10 models in the train dataset; **(B)** Performance metrics of the 10 models in the test dataset.

Figure 6 compares the outcomes of the 10 predictive models across both training and test sets. Along with excelling in AUROC, the GBM model produced commendable outcomes in the test set for Brier score (0.0259), accuracy (0.919), sensitivity (0.960), specificity (0.912), precision (0.649), and F1 score (0.774).

**Figure 7:**
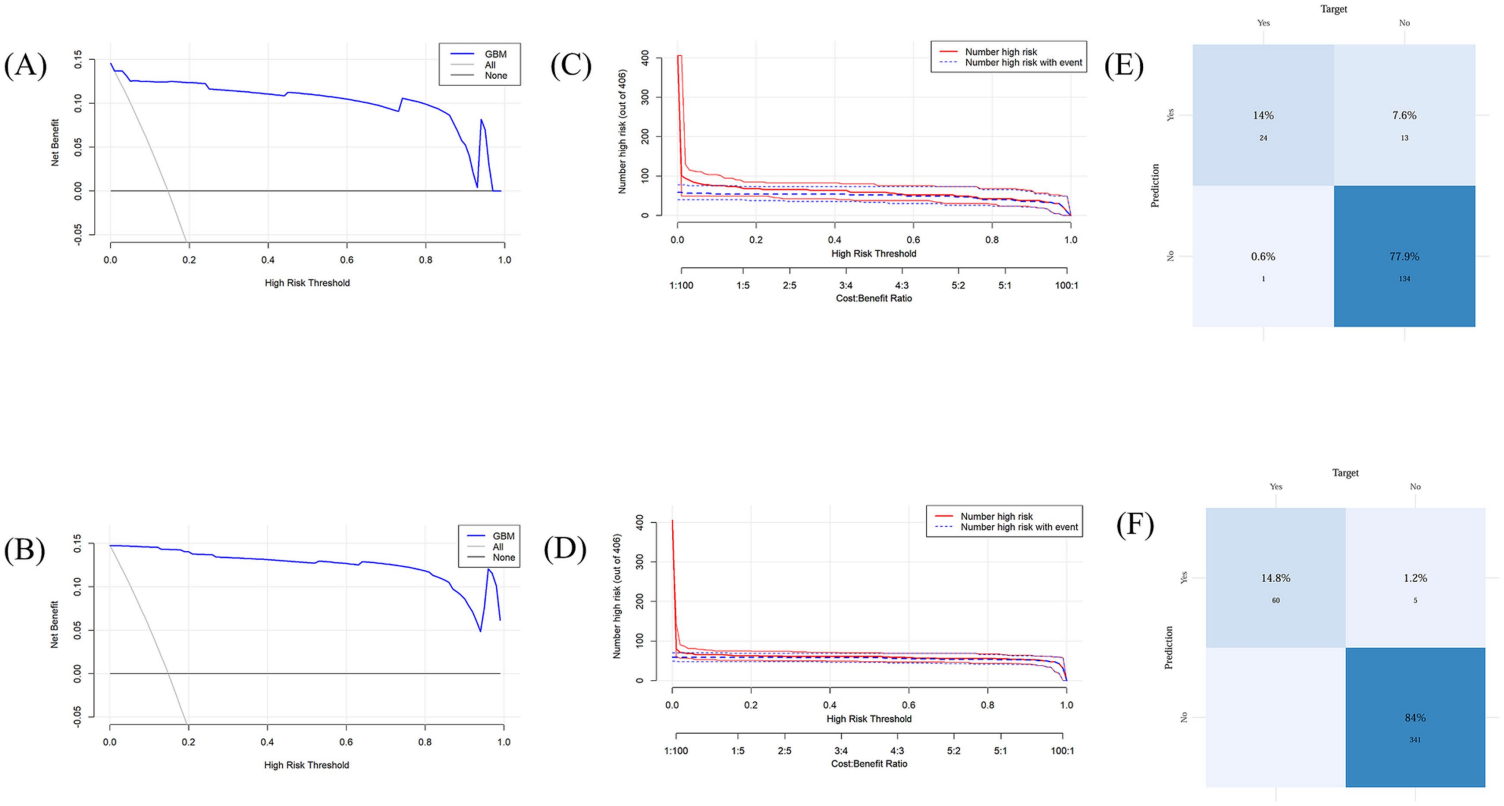
**(A)** Decision curve analysis for the test set; **(B)** Decision curve analysis for the training set; **(C)** Clinical impact curve for the test set; **(D)** Clinical impact curve for the training set. **(E)** Confusion matrix for the test set; **(F)** Confusion matrix for the training set.

The model demonstrated favorable net returns in both the training and test sets within a specific threshold probability range (Figures 7A,B). The clinical impact curve (CIC) was used to assess the clinical utility and applicability of the model (Figures 7C,D). The CIC indicated that for high-risk elderly patients with POD, when the threshold probability exceeded the prediction score, the predictive model was highly aligned with the actual population. This confirms the model’s effectiveness in clinical practice.

The training set model accurately detected 339 true negatives, 60 true positives, and 5 false positives in the confusion matrix analysis (Figures 7E,F). The sensitivity, and true positive rate, was 14.8%, while the specificity, and true negative rate, was 84%. The model accurately detected 24 true positives and 137 true negatives in the test set, along with 13 false positives and 1false negatives. There was a 77.9% true negative rate and a 14% genuine positive rate.

**Figure 8:**
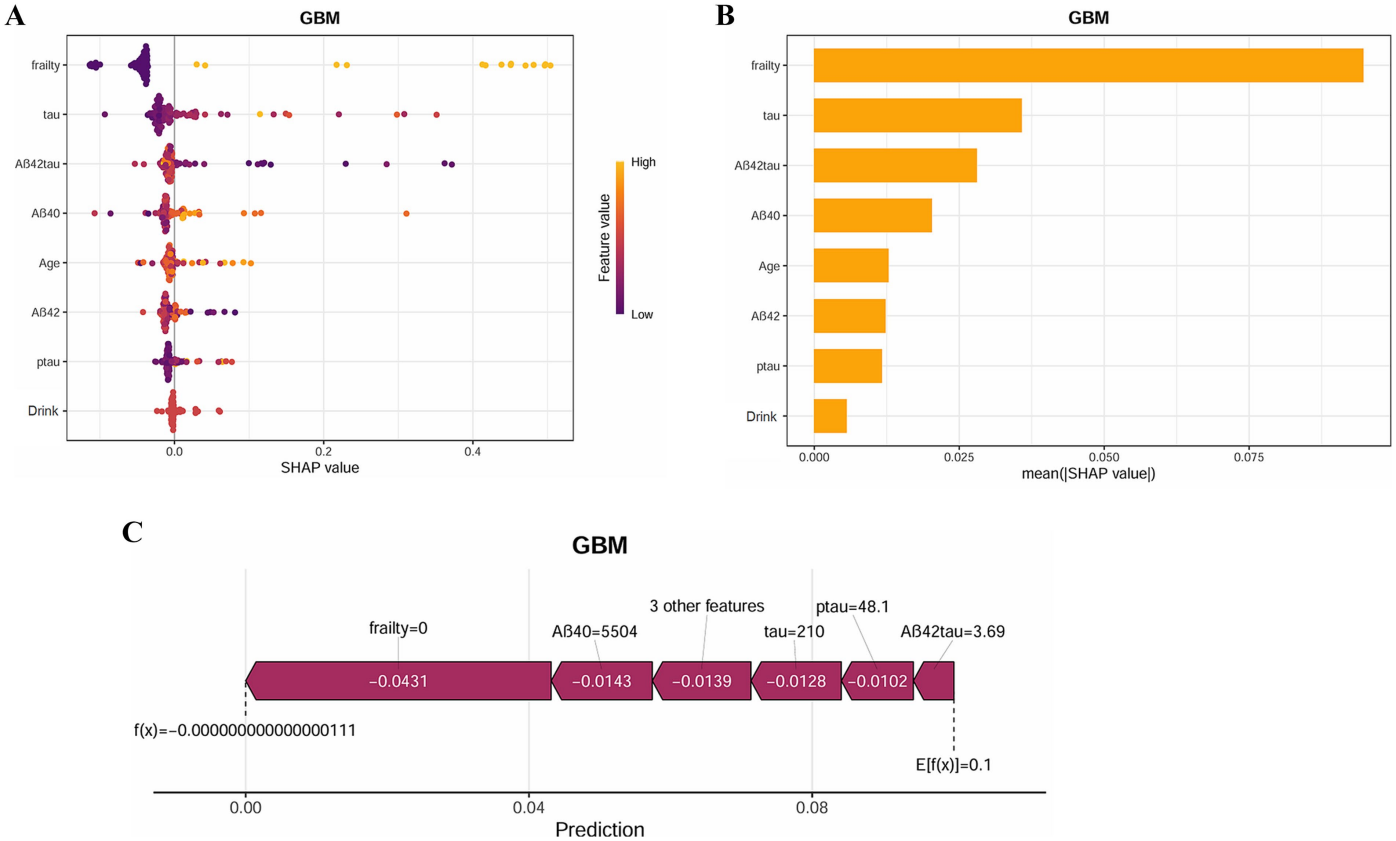
SHAP analysis provides interpretable insights for clinical decision-making. **(A)** SHAP summary plot of the GBM model. The color represents the value of the variable. **(B)** SHAP importance for each feature of the GBM model. **(C)** SHAP force plot of the GBM model.

This research investigated the primary determinants contributing to postoperative delirium in frail individuals. As illustrated in Figure 8A, the ranking of these factors is displayed, with each data point signifying an individual case, while the color gradient indicates variations in feature values. The vertical axis arranges the features based on their relative importance, simultaneously presenting their correlation with SHAP values and overall distribution. Figure 8B highlights the influence of the eight most significant predictors on forecasting outcomes. Additionally, Figure 8B further explains the hierarchical structure of feature importance in the GBM model, where the vertical axis orders the factors from most to least significant, and the horizontal axis represents their corresponding mean SHAP values. The findings reveal that Frailty and Tau hold the highest predictive relevance in assessing delirium risk. To enhance understanding of how the model makes individual-level decisions, an interpretability analysis was conducted, as shown in Figure 8C. By examining SHAP values in specific cases, the role of each factor in shaping the model’s predictions becomes evident.

## Discussion

Our findings revealed that frail patients exhibited a significantly higher incidence of POD compared with non-frail patients. Furthermore, the combination of CSF biomarkers demonstrated superior predictive power for POD. We subsequently developed a GBM-based prediction model, which showed excellent performance in internal validation and enabled early identification of POD in frail patients.

Postoperative delirium is an acute disturbance of attention and cognition, and is a common, severe, costly, and often fatal condition in the elderly (3). As life expectancy grows, so too does the number of senior citizens undergoing surgery. Delirium is the most common postoperative complication in older adults, with an incidence of 15–25% among elderly surgical patients (22). The pathophysiology of delirium remains incompletely understood and is likely driven by a range of pathogenic mechanisms. Current evidence suggests that factors such as drug toxicity, inflammation and acute stress response, blood–brain barrier disruption, amyloid-beta (Aβ) deposition, Tau phosphorylation, and neuroinflammation significantly contribute to neurotransmitter imbalances (23), playing an important role in the development of POD.

POD, a transient yet critical cognitive impairment following surgery, is strongly associated with neurodegenerative processes, in which Tau protein and its phosphorylated form (P-tau) play a pivotal role. Elevated cerebrospinal fluid Total tau (T-tau) reflects neuronal damage or axonal degeneration, while pathological tau hyperphosphorylation is a key step in neurofibrillary tangle formation. Tau’s primary function involves binding to microtubules, ensuring the stability of the microtubule network, which in turn regulates intracellular transport. In the context of neurons, microtubules are integral to the cytoskeleton, contributing to both the structural integrity of the cell and its dynamic ability to facilitate intracellular signaling and molecular trafficking (24). Abnormal T-tau loses its ability to bind to microtubules, leading to microtubule depolymerization and neuronal structure collapse. The accumulation of T-tau deposits can severely impair cognitive function, especially memory and learning. P-tau accumulates to form neurofibrillary tangles (NFTs), which physically hinder neuronal function and disrupt normal cell activity. In addition, P-tau may interfere with the PI3K/Akt signaling pathway by disrupting protein complexes located within the cell membrane. At the same time, P-tau can also inhibit the activity of PI3K through direct interaction with enzymes or their upstream regulators, thereby hindering the activation of this pathway and its downstream signaling (25).

Frailty, as a multifactorial syndrome closely associated with the aging process, is typically characterized by significant declines in physical strength, daily activity levels, nutritional status, muscle mass, and immune function. Frailty affects various bodily systems and often coexists with immune system dysfunction, metabolic abnormalities, and neurodegenerative changes. These factors may interact, collectively exacerbating the patient’s pathological condition. In this context, the relationship between frailty and POD has gradually attracted the attention of researchers. This study proposes that the potential connection between frailty and POD may be enhanced through the action of Tau protein. First, The PI3K/Akt signaling pathway is widely recognized as a key player in the development and advancement of frailty. Over time, as aging takes its toll, the function of this pathway becomes increasingly inhibited, which in turn triggers a disproportionate surge in GSK-3β activity. Activation of GSK-3β induces the phosphorylation of Tau protein at multiple sites, altering its structure, causing it to dissociate from microtubules and form aggregates (26). This process not only impairs the normal function of neurons but also interferes with intercellular signaling (27), thereby accelerating the progression of neurodegenerative changes. Additionally, abnormal phosphorylation and aggregation of Tau protein are central factors in neurodegenerative diseases. Studies have shown that Tau protein undergoes acetylation at the K15 site by GSK-3β, forming the GSK-3β K15-acetylated form. This modification inhibits the ubiquitination of GSK-3β, thereby enhancing its activity and establishing a vicious cycle (28), this cycle perpetuates Tau phosphorylation, exacerbating the formation of neurofibrillary tangles. As the process progresses, the normal function of the PI3K/Akt signaling pathway is further disrupted, accelerating neuronal death and intensifying neuronal dysfunction and signal transduction impairments (29, 30). These pathological changes provide a potential mechanism to explain the role of frailty in the development of postoperative delirium.

In recent years, machine learning, as a powerful data analysis tool, has seen increasing applications in the medical field, especially demonstrating its great potential in disease prediction and risk assessment. Risk prediction models derived from machine learning have now been developed to predict delirium and postoperative complications in hospitalized patients. In our study, we developed 10 delirium prediction models using eight variables: frailty, Tau, Aβ42/Tau, Aβ40, age, Aβ42, P-tau, and drink. Among these, the GBM model was the higher predictor. Compared to the other nine models, GBM achieved the higher AUC values in both the training and testing sets, 99.9 and 97.4%, respectively, and demonstrated better sensitivity and specificity.

We performed a comprehensive evaluation of the GBM model’s performance on the training and test sets, resulting in decision curve analysis (DCA) curves. The results showed that the GBM model provided significant net benefit within specific probability threshold ranges, indicating its utility in clinical applications. To further validate its clinical applicability, we analyzed the clinical impact curve and confusion matrix, which confirmed the advantages of the GBM model, particularly in improving diagnostic accuracy and risk assessment precision. This demonstrates its strong advantages and practical value in real-world clinical settings.

This study employed the SHAP method to offer both global and individual-level insights into the GBM model, further improving its visualization and transparency. By applying SHAP analysis, we were able to identify the precise roles that each attribute played in the model’s predictions, thus bolstering its interpretability. This technique facilitated a deeper understanding of how the model makes decisions, which in turn increases its reliability and suitability for clinical applications.

### Limitations

This study has several limitations. First, the cost and accessibility of CSF biomarker testing pose significant challenges for clinical implementation. Second, the study included a limited number of variables, and incorporating preoperative blood inflammatory markers could further enhance the predictive model’s comprehensiveness. Third, POD assessment primarily used the MDAS by trained staff; however, retrospective review of medical records when assessors were unavailable may have introduced bias due to incomplete documentation, potentially underreporting mild delirium cases. Forth, the exceptionally high AUC (0.983) for frailty combined with CSF biomarkers raises concerns about overfitting, especially given the single-center sample. External validation in multi-center cohorts is essential before clinical implementation. Finally, external validation and integration of cost-effective biomarkers (e.g., blood-based inflammatory markers) are needed to enhance clinical utility.

## Conclusion

In summary, the relationship between frailty and postoperative delirium offers new perspectives. By monitoring cerebrospinal fluid biomarkers and applying machine learning models, we can more accurately predict the risk of postoperative delirium. The GBM model used in this study demonstrates excellent performance in predicting postoperative delirium by fully exploring the underlying relationships within the data. Future research could further integrate more clinical data and other types of biomarkers to optimize the predictive model, enhance prediction accuracy, and enhance postoperative delirium prevention and management in clinical settings.

## Data Availability

The original contributions presented in the study are included in the article/supplementary material, further inquiries can be directed to the corresponding author.
